# Periodontal Disease in Obese Patients; Interleukin-6 and C-Reactive Protein Study: A Systematic Review

**DOI:** 10.3390/dj10120225

**Published:** 2022-11-29

**Authors:** Julieta Cruz-Ávila, Elizabeth Hernández-Pérez, Rogelio González-González, Ronell Bologna-Molina, Nelly Molina-Frechero

**Affiliations:** 1 Dental Sciences, Department of Health Care, Universidad Autónoma Metropolitana Xochimilco (UAM-X), Mexico City 04960, Mexico; 2 Department of Health Sciences, Universidad Autónoma Metropolitana Iztapalapa (UAM-I), Mexico City 09340, Mexico; 3 Department of Research, School of Dentistry, Juarez University of the Durango State (UJED), Durango 34000, Mexico; 4 Molecular Pathology Area, School of Dentistry, University of the Republic (UDELAR), Montevideo 11200, Uruguay; 5 Department of Health Care, Universidad Autónoma Metropolitana Xochimilco (UAM-X), Mexico City 04960, Mexico

**Keywords:** IL-6, periodontal disease, obesity, C-reactive protein

## Abstract

Periodontal disease (PD) and obesity are characterized by a dysregulated inflammatory state. Both conditions trigger inflammatory and immune responses with an increase in proinflammatory cytokines such as Interleukin 6 (IL-6) and the release of inflammatory mediators such as C-reactive protein (CRP). Individuals with a high body mass index (BMI) present a chronic inflammatory state. The aim of the present study was to perform a systematic review of inflammatory markers (IL-6 and CRP) in obese patients with PD and their possible relationship by analyzing the levels of these markers. A digital literature search was performed in three databases—PubMed, SciElo and Medigraphic—through an advanced search for original articles, employing IL-6 and CRP in obese patients with PD, within a publication period from 2010 to 2021. PRISMA guidelines, the JADAD scale and a qualitative analysis of scientific evidence were performed using the Cochrane collaboration method and the RoB 2 assessment tool. Ten articles were included in this analysis with the variables recorded and associated with subjects with obesity and PD. Of the ten articles included, three analyzed IL-6 and CRP, four analyzed IL-6 and three analyzed CRP. In conclusion, and based on the available evidence, the aforementioned markers of inflammation demonstrate that there is a relationship between PD and obesity.

## 1. Introduction

Periodontal disease (PD) represents a group of diseases localized in the gingiva and supporting structures of the tooth, which are produced by certain bacteria from the subgingival plaque and constitute the main cause of tooth loss in adults [1,2,3]. In particular, it is characterized by inflammation that extends deep into the tissues and causes the degeneration and destruction of the supporting tissue and alveolar bone [4,5].

Clinically, it is characterized by attachment loss detected at two or more non-adjacent interproximal sites or an attachment loss of 3 mm or more on the vestibular or palatal/lingual sides in at least two teeth not caused by the following: (1) gingival recession of traumatic origin, (2) dental caries extending into the cervical region of the tooth, (3) attachment loss on the distal aspect of a second molar and a direct association with second molar malposition or third molar extraction, (4) endoperiodontal lesions that drain through the marginal sulcus, or (5) root fracture of a vertical type [6].

Within the new classification, periodontitis can be classified with stages/stages and grades (classification of periodontal and peri-implant diseases and conditions 2018) [7,8].

Stages classify the severity and extent of tissue loss, including tooth loss due to periodontitis, and incorporate an assessment regarding the complexity of the long-term management of the patient’s function and esthetics. Therefore, the severity of the disease and complexity of management are divided into four stages: Stage I—Initial Periodontitis; Stage II—Moderate Periodontitis; Stage III—Severe Periodontitis, with the potential for additional tooth loss; Stage IV: Severe Periodontitis with potential for loss of the dentition [9]. Grade reflects the evidence or risk of disease progression and its effects on systemic health. Grades: evidence or risk of rapid progression; anticipated response to treatment: Grade A: slow progression, Grade B: moderate progression, Grade C: rapid progression [10].

The pathophysiological mechanism by which this process occurs is explained by the host immune response to toxin-producing microorganisms. These endotoxins stimulate periodontal tissue defense cells to express different inflammatory mediators [5]. These responses are not only limited to periodontitis but can also be seen in systemic conditions such as diabetes, arthritis and obesity [11].

Obesity is a chronic, multifactorial disease and is the most common nutritional disorder [12]. It is defined as the excessive accumulation or abnormal distribution of fat in the body with health consequences [13]. A classification accepted by the WHO and widely used, but with great limitations, is the body mass index (BMI, kg/m^2^) [14,15]. In adults, a BMI of 25 to 29.9 kg/m^2^ is defined as overweight, while a BMI > 30 kg/m^2^ is defined as obese [16]. The classification for obese subjects is as follows: Grade 1 obesity with a BMI of 30–34.5; Grade II between 34.5 and 40; and Grade III > 40 kg/m^2^ [14,15].

### 1.1. Hormones

Adiponectin is involved in lipid and glucose metabolism in insulin-sensitive tissues. This hormone increases insulin sensitivity, reduces glucose production, and stimulates fatty acid oxidation. Plasma adiponectin concentrations decrease with insulin resistance (as occurs with type 2 diabetes). Low levels of this hormone are related to obesity [16].

### 1.2. Obesity-Related Diseases

Patients with obesity are at risk of increased morbidity from diverse diseases, including dyslipidemia, type 2 diabetes mellitus, hypertension, coronary heart disease, respiratory problems, sleep apnea, some types of cancers and PD. Subjects with a BMI > 40 kg/m^2^ significantly increase their risk for diabetes, hypertension, hyperlipidemia, asthma and arthritis [17].

In obesity, an increase in adipose tissue is observed, which translates into an increase in BMI. Adipose tissue shows an elevated production and secretion of a variety of proinflammatory molecules, such as Tumor Necrosis Factor-α, Interleukin-6, Interleukin-8 and C-reactive protein, which may have local effects on adipose cell physiology and systemic effects on other organs [12].

The adipose tissue releases adipose cells, which include adipocytes and macrophages that secrete more than 50 adipokines and cytokines, including adiponectin, leptin [5], TNF-α [18], visfantin and resistin [19], among others [20], which impact local and systemic metabolism and inflammation.

Adiponectin modulates a number of metabolic processes, such as glucose regulation, blood pressure and fatty acid catabolism, and has inverse associations with serum markers of inflammation [1]. Leptin, which was the first adipokine to be studied, is involved in temperature regulation and appetite control [21]; in obese patients, it is found in increased levels, suggesting leptin resistance [22]. Some of these hormones act locally and others are released into systemic circulation and function as signaling molecules in the liver, muscle, and the endothelium [23,24]. In the inflammatory processes of obesity and PD, the concentration of inflammatory markers increases; one of these markers is C-reactive protein (CRP). These are serum proteins called acute phase reactants [12], which are synthesized in the liver during the acute phase of the immune response and are normally present in serum at trace levels with concentrations of less than 0.3 mg/dL [25]. C-reactive protein and other proteins originate secondary to the stimulus of cytokines such as IL-6, IL-1, TNF-, interferon- (IFN-) among others [26,27].

Thus far, studies have examined different markers of inflammation that could be involved in the development of PD in patients with obesity. These studies focused on evaluating the values found in IL-6 and CRP, which could establish a possible link between these two diseases [28,29].

Therefore, the aim of the present study was to perform a systematic review of the relationship between PD and obesity based on the values of the inflammation markers IL-6 and CRP detected in previously studied individuals.

## 2. Methods

### 2.1. Protocol and Registration

The literature related to PD and obesity was reviewed based on PRISMA [30] guidelines and was previously registered in the Prospective International Registry of Systematic Reviews (PROSPERO) [31] database, with registration number CRD4202022315418.

### 2.2. Search and Selection of Evidence

A digital literature search was performed in the main databases—PubMed, SciElo, Medigraphic—using medical subject headings (MeSH) and terms related to PD and obesity. The keywords used for the advanced search in each database were: IL-6, Periodontal disease, obesity, C-reactive proteins, together with the Booleans AND and OR, using the following terms without abbreviations: “IL-6 AND periodontal disease”, “Periodontal disease AND Obesity”, “Obesity AND IL-6” OR “C reactive proteins”, “C reactive proteins AND Obesity OR Peridontal disease”.

In the initial process, articles that connected PD with obesity were identified, eliminating duplicates. In the filtering phase, articles were excluded by title and abstract; in the eligibility phase, the full text was reviewed and articles that did not correspond to the scope of the review were excluded; and in the inclusion phase, articles were selected for the final analysis.

Search results were cross-checked to eliminate duplicates. Two researchers used Microsoft Excel to independently extract the data in order to compare the recorded information and correct the differences found during this step according to the proposed variables: a third reviewer intervened in the case of disagreement between the reviewers, to resolve the problem, and finally, both reviewers filled in the forms of each study again with the corrected information.

### 2.3. Eligibility Criteria

Articles were selected that met the following inclusion criteria: articles that were published in the period 2010–2021, original studies conducted on humans, clinical trials that evaluated the relationship between PD and obesity and studies with participants over 18 years of age. The exclusion criteria included records related to PD associated with other systemic diseases and other subareas of dentistry, articles that evaluated any medication, studies carried out on pregnant women, articles discussing patients with inflammatory and periodontal treatments prior to the experimental study, articles that did not meet the requirements for inclusion and those with irrelevant content.

### 2.4. Data Elements

The included and analyzed variables were: year of publication of the study, study site, study design, number of participants, sex, age range of the participants, inflammation marker values: IL-6 and CRP and BMI and PD values. These were sufficient and adequate for obtaining relevant data to demonstrate the possible relationship between obesity and PD.

### 2.5. Assessment of Methodological Quality and Risk of Bias

For the evaluation of the methodological quality of the articles included in this review, the JADAD scale was used [32]; this procedure is used to independently evaluate a clinical trial. It has a score range of 0 to 5 points. A clinical trial with a score greater than three is considered rigorous and of poor quality if the score is less than three points. The questions of this scale were asked to each article giving one point for each answer.

The Cochrane collaboration tool was used to assess the risk of bias in the selected studies, which includes the following domains: risk of bias due to confounding, risk due to participant selection, risk due to loss of data, risk in the measurement of results, risk in the selection of results and risk of general bias.

Each domain was given a specific rating using the terms “low risk”, “unclear”, and “high risk”. The Risk-of-bias VISualization (robvis) tool was used to produce the risk-of-bias plots (Cochrane and robvis) [33]. The two assessments were performed by two independent reviewers, and a third reviewer was involved to resolve any disagreements.

### 2.6. Summary of Measures

The measures included were the standard deviation (SD) of each immunological marker (IL-6 and CRP) analyzed, and the *p* value to support significant correlations of the reported results with respect to the serum levels of these markers in groups of people with PD and obesity compared to the control groups.

### 2.7. Synthesis of the Results

The data from each study analyzed were recorded in a database using Microsoft Excel software and organized according to the variables of interest in order to simplify the interpretation and comparison of the results.

## 3. Results

After the evaluation of the methodological quality based on the JADAD Scale criteria, the ten chosen articles were found to be of rigorous quality with scores higher than three points (Table 1).

### 3.1. Selection of Studies

**Figure 1:**
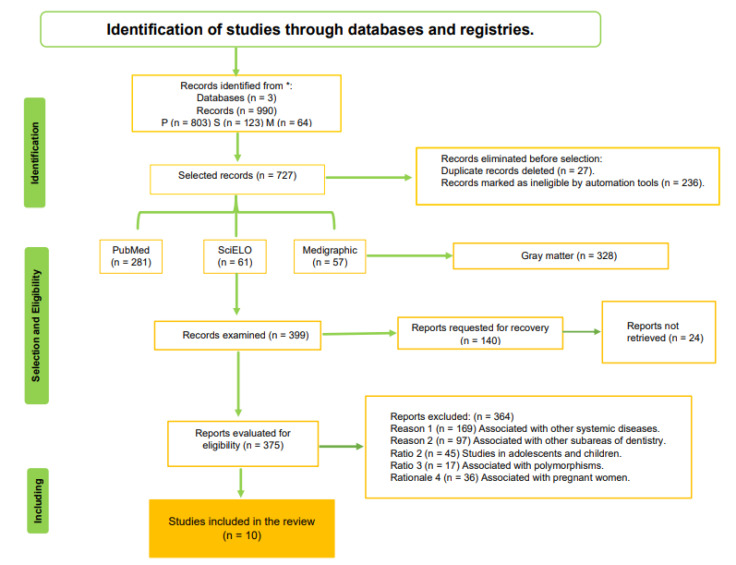
PRISMA flow diagram for the systematic review. Of the 990 articles found in the three databases included in the search, 10 studies were selected for analysis in this study.

A total of 990 articles were found in the electronic search. Of these, 263 were eliminated before selection and marked as ineligible by automation tools because they were duplicate records. Subsequently, 328 articles were eliminated because they corresponded to thesis works, book chapters, memoirs, projects, among others. Furthermore, 399 articles were evaluated in abstract and titles, and 140 articles were requested for retrieval, 24 of which could not be retrieved. We were left to determine the eligibility of 375 articles, and 364 of them were excluded for different reasons. According to our selection criteria, 10 articles were included in the systematic review after a thorough evaluation. Figure 1 shows the PRISMA flow chart with the process of evaluation and selection of the chosen studies.

Table 2 shows the general data of the articles that were included in this review: 40% were carried out in Turkey, 30% in Brazil and 10% in Chile, Thailand and China. In total, 70% percent of the studies were case–control, 10% were cohort studies, 10% were randomized double-blind trails and 10% observational studies. The journals of publication and the inflammatory markers that were analyzed in each study are also shown, of which 30% analyzed IL-6 and CRP, 40% IL-6 and 30% CRP.

### 3.2. Assessment of Risk of Bias of the Studies

**Figure 2:**
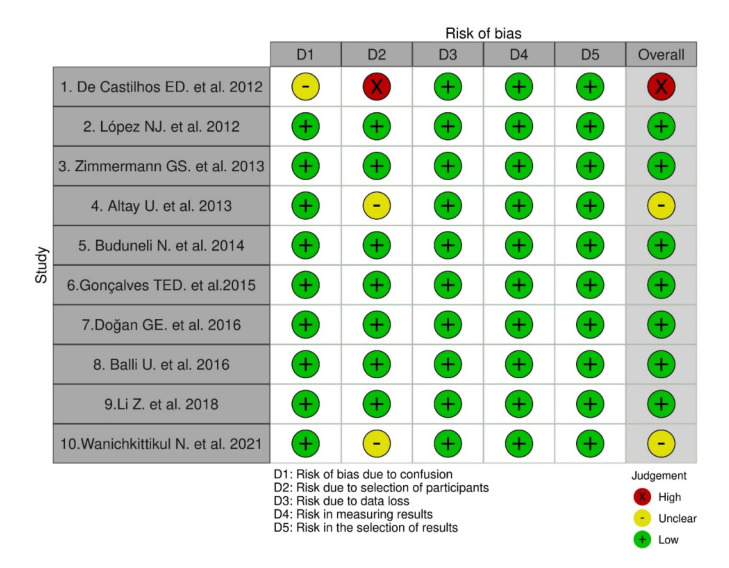
Risk of bias for each of the articles included.

**Figure 3:**
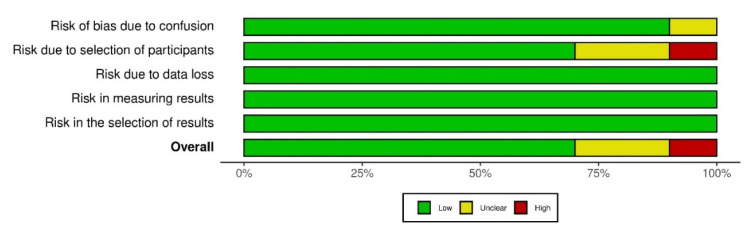
Risk of bias according to each domain and overall risk of bias.

Seven studies (70%) had a low risk of bias, followed by two studies with an unclear risk (20%) and one study showed a high risk (10%) (Figure 2). The results obtained from the risk of bias analysis in the individual studies according to the evaluated domains are shown. The overall risk of bias was over 70%, with all the articles at low risk of bias (Figure 3). Given the results of the risk of bias analysis, the content of the selected articles was relevant for the development of the present review.

### 3.3. Characteristics of the Study

A total of 774 research subjects participated in the ten included articles, of which 506 were cases and 268 controls. There were 478 (60%) female participants, while 325 (40%) were male; the age range of the participants was 23 to 65 years (Table 3).

All the articles studied used the ELISA (enzyme-linked immunoadsorbent assay) technique for the analysis of samples of inflammatory markers (IL-6 and CRP). These samples were taken from serum, saliva and FCG (gingival crevicular fluid). Of the total number of samples, 60% of the articles used serum samples, 10% saliva and FCG and 20% used both serum and FCG (Table 4).

Table 5 shows the ten studies in which IL-6 values, CRP or both were measured in patients with PD and obesity compared to control groups. The stage of PD and the degree of obesity found in each study group is also shown. The data obtained in the different studies show a significant increase in IL-6 and CRP values in the PD and obesity groups compared to the control groups, finding a positive correlation with PD and obesity.

## 4. Discussion

Based on the data obtained from the articles included in this systematic review, we found an association between PD and obesity. The latter may influence PD status by increasing the levels of IL-6 and CRP [43].

The mechanisms responsible for the association between PD and obesity have not yet been described. No controlled trial studies have obtained specific evidence of this association; however, in recent years, several cohort studies have confirmed the association between PD and obesity. One of the proposed mechanisms derives from the fact that pathological oral bacteria can induce traumatic damage causing irritation of the epithelium and mucosa, and thus play an important role in the subsequent progression, not only in the correlation with obesity but also in lifestyle-related comorbidities. Furthermore, it is suggested that chronic, localized inflammation induces systemic inflammation, leading to endothelial disruption [44].

Inflammation plays an important role in local and systemic pathogenesis. One hypothesis indicates an increase in proinflammatory cytokines from the gingival fluid (GCF), serum and saliva in obese subjects [36,37]. This increase is enhanced by the chronic state of inflammation present in obese individuals, which in turn is increased by the secretion of adipokines, such as leptin and adipokine, from adipocytes [45].

Adipose tissue was originally considered as an inert tissue, only with the function of storing fat. However, today, this tissue is known to have an effective metabolic activity derived from the action of several cytokines and adipokines. Cytokines secreted by adipocytes, such as TNF-α and IL-6, cause alterations in glucose metabolism leading to an increase in glucose secretion. This is correlated with increased BMI and adipocyte size [34,43,44,45,46,47].

Overweight and obesity have become a public health problem, not only because of the various comorbidities with which they are associated, but also because of their economic impacts. Obesity of a multifactorial nature has been linked to different diseases such as asthma, sleep apnea, arthritis, some types of cancer, and non-alcoholic liver disease, among others, and periodontal disease is no exception from this list. Perlstein et al., 1977 [46], were the first researchers to report an association between obesity and PD, and several epidemiological and meta-analysis studies support the idea that obesity is a risk factor for PD [29,47]. In this regard, several authors have found an association between one or more periodontal parameters in obese patients compared with patients of a normal weight [39,42,48].

Corroborating the hypothesis of an association between PD and obesity, Lee J. H et al., 2020 [44], found an association with PD and obesity, in this report the authors performed a retrospective study with a cohort of 558,000 patients analyzing the relationship of PD with lifestyle-related comorbidities, finding a significant association with obesity, dyslipidemia and hypertension. Similar results were found by Ngoude J.X.E et al., 2021 [49], the authors analyzed the relationship between PD and metabolic syndrome, finding a significant association with obesity and low HDL levels.

Obesity is characterized by fatty acid storage and adipose tissue extension, associated with the development of insulin resistance in peripheral tissues such as skeletal muscle and the liver. The expansion of adipose tissue in obesity leads to increased macrophage infiltration and inflammation with an increased production of proinflammatory cytokines such as TNF-a and IL-6. This is accompanied by an increased release of free fatty acids and the altered secretion of adipokines such as leptin [50]. One study has reported an association between PD severity and leptin levels in serum and GCF [51].

Leptin was the first adipokine described with a molecular weight of 16 k Da; it is a non-glycosylated peptide. It is mainly produced by fat cells, placenta, T cells, osteoblasts, and gastric epithelial tissue. In addition to regulating food intake, energy flow and lipid and bone metabolism, leptin can also regulate the immune inflammatory process [50]. Several studies suggest that leptin plays an important role in the development of PD, as it is significantly increased in the serum of obese patients as well as periodontal parameters such as clinical attachment loss and probing depth [21]. In line with these data, Altay U et al., 2013 [38], reported that leptin serum values were significantly associated with PD severity, indicated by the percentage of positive bleeding on probing (BOP) sites, while the results obtained by Li Z et al., 2018 [41], revealed a significant association between leptin and resistin with CRP and IL-6, via a Spearman’s analysis, thus indicating an association between obesity and PD.

Contrary data were reported by Budenelli, N et al., 2014 [39], who found no association between periodontal parameters and obesity even though they presented significant increases in IL-6 and leptin in obese versus normal weight subjects (*p* < 0.05). Similar results were reported by Gonçalves T.E et al., 2015 [22], where they indicated that obesity might modulate the systemic and periodontal levels of adipokines in favor of proinflammation.

Another mechanism that may explain the link between PD and obesity is systemic inflammation. As observed by an increase in the serum levels of C-reactive protein and other biomarkers derived from PD, this pathway explains the relationship between these two pathologies and the relationship between PD and lifestyle-related comorbidities, such as diabetes mellitus, hypertension and dyslipidemia among others [44].

C-reactive protein is an acute phase protein expressed in response to systemic inflammation, since obesity can trigger an increased local inflammatory response to an external stimulus, such as dental plaque [35]. In this sense, De Castillos E.D et al., 2012 [34], reported an association with obesity and PD, which is mediated by an increase in CRP. Based on their results, the authors indicate that this association is mediated by systemic inflammation, suggesting that the risk of presenting gingival bleeding is associated with increased BMI. Similar results were reported by Li Z et al., 2018 [41]; these authors found a significant increase in CRP and IL-6. They also found an increase in other cytokines, such as IL-1β and TNF-α. Contrary data were reported by Wanichkittikul N et al., 2021 [42], and Altay U et al., 2013 [38]. These authors found no significant differences between obese patients with and without PD in CRP values, even though their results presented significant differences in periodontal parameters, likely because of the n of their data.

Obesity is associated with a state of inflammation that may trigger the initiation of chronic noncommunicable diseases as previously discussed, and this may be related to the severity of the PD [46]. High amounts of adipose tissue can exacerbate the local and systemic inflammatory system, and thus can establish or aggravate disorders of an inflammatory nature such as gingivitis [34]. Gingivitis is characterized by an inflammatory response; inflammatory molecules such as IL-6 and anti-inflammatory molecules such as IL-10 may be altered in saliva [51,52].

Obesity and PD, either independently or together, can alter local and systemic levels of adipokines, mainly targeting proinflammation [53]. Furthermore, leptin and IL-6 were associated with individuals with obesity and PD. This derives from data obtained by Zimmermann G.S et al., 2013 [36], who found alterations in adipokine levels at the serum level and in GCF in obese individuals with periodontitis, compared to the control group of normal weight patients without PD. However, the hypothesis that individuals with obesity and periodontitis have the highest values of proinflammatory cytokines and adipokines was rejected since their study groups showed significant differences in age between the obese and normal weight individuals.

## 5. Conclusions

It is possible to determine, based on the available evidence, that there is a relationship between PD and obesity. The relevance of these findings centers on the high levels of expression of inflammatory factors and overexpressed adipocytokines that may be important factors in persistent obesity and in the development or worsening of periodontal disease. Obesity and PD cause impaired glucose tolerance, alterations in the host immune system, an increase in and activation of macrophages, altered microvascular function and the secretion of proinflammatory substances from adipose tissue. This tissue releases proinflammatory cytokines such as: TNF-α, IL-6, among others, and acute phase proteins (CRP). These findings provide preliminary information and biological explanations for the clinical evidence that obesity affects PD progression.

## 6. Limitations

One of the limitations of this review is that no manuscript was found that demonstrated the mechanisms by which obesity is linked to periodontal disease. We found diverse proposals that describe the link between these two diseases; however, these proposals are merely presented as hypotheses. Likewise, the number of used items should have been larger.

Similarly, we included many markers of inflammation that were different from the ones we chose to analyze, which is another limitation to our review. Nevertheless, our results were as expected.

## Figures and Tables

**Table 1:** JADAD scale for assessing the overall quality of articles.

AUTHOR, YEAR	Was the Study a Controlled Experimental Study?		Was There a Clear Description of the Inclusion and Exclusion Criteria?		Was the Method Used in the Clinical Trial Adequate?		Was There at Least One Control (Comparison) Group?		Were the Statistical Analysis Methods Described?		Were the Outcome Measures Clearly Defined?		Another Bias		JADAD Scale	Methodological Quality
	Yes = 1	Not = 0	Yes = 1	Not = 0	Yes = 0	Not = −1	Yes = 0	Not = −1	Yes = 1	Not = 0	Yes = 1	Not = 0	Yes = 1	Not = 0	Points	Good Quality +3 Poor Quality −3
1. De Castilhos ED et al., 2012 [34]	1			0	0			−1	1		1		1		3	Good quality
2. López NJ et al., 2012 [35]	1		1		0		0		1		1		1		5	Good quality
3. Zimmermann GS et al., 2013 [36]	1		1		0		0		1		1		1		5	Good quality
4. Doğan GE et al., 2016 [37]	1		1		0		0		1		1		1		5	Good quality
5. Altay U et al., 2013 [38]	1		1		0			−1	1		1		1		4	Good quality
6. Buduneli N et al., 2014 [39]	1		1		0		0		1		1		1		5	Good quality
7. Gonçalves TED et al., 2015 [22]	1		1		0		0		1		1		1		5	Good quality
8. Balli U et al., 2016 [40]	1		1		0		0		1		1		1		5	Good quality
9. Li Z et al., 2018 [41]	1		1		0		0		1		1		1		5	Good quality
10. Wanichkittikul N et al., 2021 [42]	1		1		0		0	−1	1		1		1		4	Good quality

+: Greater than three points, −: Less than three points.

**Table 2:** General Summary of the Selected Articles.

Author, Year	Country	Type of Study	Article Title	Journal of Publication	Inflammation Marker
1. De Castilhos ED et al., 2012 [34]	Brazil	Cohort	Association between obesity and periodontal disease in young adults: a population-based birth cohort.	J. Clin. Periodontol.	CRP
2. López NJ et al., 2012 [35]	Chile	Randomized double blind	Effects of periodontal therapy on systemic markers of inflammation in patients with metabolic syndrome: a controlled clinical trial.	J. Periodontol.	PCR
3. Zimmermann GS et al., 2013 [36]	Brazil	Cases and controls	Local and circulating levels of adipocytokines in obese and normal weight individuals with chronic periodontitis.	J. Periodontol.	IL-6
4. Doğan GE et al., 2016 [37]	Turkey	Cases and controls	Salivary IL-6 and IL-10 levels in subjects with obesity and gingivitis.	Am. J. Dent.	IL-6
5. Altay U et al., 2013 [38]	Turkey	Cases and controls	Changes in inflammatory and metabolic parameters after periodontal treatment in patients with and without obesity.	J. Periodontol.	IL-6 CRP
6. Buduneli N et al., 2014 [39]	Turkey	Cases and controls	Is obesity a possible modifier of periodontal disease as a chronic inflammatory process? A case-control study.	J. Periodontal. Res.	IL-6 CRP
7. Gonçalves TE et al., 2015 [22]	Brazil	Cases and controls	Local and serum levels of adipokines in patients with obesity after periodontal therapy: one-year follow-up.	J. Clin. Periodontol.	IL-6
8. Balli U et al., 2016 [40]	Turkey	Cases and controls	Chemerin and interleukin-6 levels in obese individuals following periodontal treatment.	Oral Dis.	IL-6
9. Li Z et al., 2018 [41]	China	Cases and controls	Correlation of serum adipocytokine levels with glycolipid metabolism and inflammatory factors in obese patients with periodontal disease.	Int. J. Clin. Exp. Pathol.	IL-6 CRP
10. Wanichkittikul N et al., 2021 [42]	Thailand	Observational	Periodontal Treatment Improves Serum Levels of Leptin, Adiponectin, and C-reactive Protein in Thai Patients with Overweight or Obesity.	Int. J. Dent.	CRP

PCR: C-reactive protein.

**Table 3:** Demographic Characteristics of the Patients.

Articles, Year	Sex (M/F)	Age Range	Cases	Controls	Total Population
1. De Castilhos ED et al., 2012 [34]	36/22	23	58	0	58
2. López NJ et al., 2012 [35]	46/119	35–65	82	83	165
3. Zimmermann GS et al., 2013 [36]	21/57	31–65	58	20	78
7. Doğan GE et al., 2016 [37]	19/21	20–55	20	20	40
4. Altay U et al., 2013 [38]	14/32	>25	22	24	46
5. Buduneli N et al., 2014 [39]	0/91	>41	60	31	91
6. Gonçalves TE et al., 2015 [22]	21/19	>30	20	20	40
8. Balli U et al., 2016 [40]	41/39	30–49	60	20	80
9. Li Z et al., 2018 [41]	126/50	<18	126	50	176
10. Wanichkittikul N et al., 2021 [42]	6/23	>35	22	7	29

M: Male, F: Female, Age range in years.

**Table 4:** Type of Sample used in each Study for Analysis.

Sample Type Used	Number of Items	Data in Percent (%)
Serum	6	60%
Salivary	1	10%
GCF	1	10%
GCF and Serum	2	20%

**Table 5:** Studies Carried out With Different Markers of Inflammation using the ELISA Method.

Author, Year	Type of Sample	Periodontal Disease	BMI (kg/m²) ± SD	Inflammation Marker ± SD	Results
Zimmermann GS et al., 2013 [36]	GCF Serum	Stage III	Degree of obesity I EG: 33.2 ± 2.8 CG: 23.4 ± 2.0	IL-6 FCG: EG: 0.47 ± 2.01 CG: 0.63 ± 1.10 Suero: EG: 3.4 ± 1.6 CG: 2.8 ± 2.3	PP: elevated in obese group (*p* < 0.05). GCF and Serum: Logistic regression analysis shows serum IL-6 levels (*p* = 0.04) correlated with obese group individuals. Relationship + between IL-6 level and BMI.
Gonçalves TE et al., 2015 [22]	GCF Serum	Stage III	Degree of obesity II EG: 36.1 ± 3.1 CG: 23.4 ± 1.0	IL-6 FCG: EG: 0.3 ± 0.7 CG: 0.3 ± 0.6 Suero: CG: 2.2 ± 0.9 EG: 2.7 ± 1.6	PP: elevated in obese group (*p* < 0.05). GCF and serum: elevated IL-6 values at 3-month follow-up (*p* < 0.05). Relationship + between IL-6 level and BMI.
Doğan GE et al., 2016 [37]	Salivary	Stage II	Degree of obesity I EG: 34.4 ± 3.2 CG: 22.9 ± 2.2	IL-6 EG: 13.9 ± 11.6CG: 6.1 ± 8.3	PP: There were no significant differences (*p* = 0.265), IL-6 levels were higher in obese subjects (*p* = 0.002). Relationship + between IL-6 level and BMI (*p* = 0.020).
Balli U et al., 2016 [40]	GCF	Stage III	Degree of obesity I EG: 33.80 ± 2.11 CG: 22.75 ± 1.29	IL-6 EG: 0.506 CG: 0.369	PP: elevated in obese group (*p* < 0.05). IL-6 levels were elevated with obesity (*p* < 0.008). Relationship + of IL-6 with BMI and clinical attachment levels (*p* < 0.05).
De Castilhos ED et al., 2012 [34]	Serum	Stage II	Degree of obesity I 1, 49 (0, 74; 3, 00)	PCR 1.23 (0.26; 5, 85)	PP: elevated in obese group (*p* < 0.05). The increase in CRP levels had a + correlation with obesity (*p* < 0.05).
López NJ et al., 2012 [35]	Serum	Stage II	Degree of obesity I EG: 29.96 ± 3.89 CG: 30.39 ± 4.26	PCR EG: 4.43 ± 3.05 CG: 04.39 ± 3.17	PP: There were no significant differences in the groups (*p* > 0.05). Elevated CRP levels in both study groups (*p* < 0.05).
Wanichkittikul N et al., 2021 [42]	Serum	Stage IV	Degree of obesity I EG: 25.07 (23.96; 29.20) CG: 19.98 (18.14, 23.32)	PCR EG: 3, 17 (2, 08; 8, 04) CG: 1, 58 (0, 66; 3, 97)	PP: Higher levels in the obese group (*p* < 0.001). CRP levels were higher in patients in the obese group (*p* < 0.001).
Altay U et al., 2013 [38]	Serum	Stage III	Degree of obesity I 13 (59.1) Degree of obesity II 6 (27.3) Degree of obesity III 3 (136)	IL-6 EG: 1.1 (0.8–1.9) CG: 1 (0.9–1.04) PCR EG: 3.3 (3.2–6.0) CG: 3.3(3.0–4.2)	PP: elevated in favor of the obese group (*p* < 0.01). No significant differences in inflammation markers.
Buduneli N et al., 2014 [39]	Serum	Stage II	Degree of obesity II (35–39.9) EG: 37.90 ± 4.56 CG: 23.00 ± 0.87	IL-6 EG: 0.59 ± 0.16 CG: 0.10 ± 0.01 PCR EG: 2.69 ± 1.81 CG: 3.19 ± 2.19	PP: clinical attachment level was significantly higher in the obese group (*p* < 0.05). Serum IL-6 levels were significantly higher in the obese group (*p* < 0.05). Serum CRP levels were similar in both study groups (*p* > 0.05). The BMI was related to elevated IL-6 levels (*p* < 0.05).
Li Z et al., 2018 [41]	Serum	Stage II	Degree of obesity I EG: 2.10 ± 0.34CG: 0.56 ± 0.08	IL-6 EG: 2.94 ± 0.61 CG: 0.56 ± 0.08 PCR EG: 8.81 ± 3.27 CG: 4.35 ± 2.04	PP: elevated in obese group (*p* < 0.05). Elevated levels of IL-6 and CRP in obese group (*p* < 0.05). The obesity status of the experimental group was related + with elevated serum levels of IL-6 and CRP (*p* < 0.05).

GCF: Gingival Crevicular Fluid; EG: Experimental Group; CG: Control Group; BMI: Body Mass Index; P.D: Periodontal Disease; CRP: C-reactive Protein; SD: Standard Deviation; PP: Periodontal Parameters; DO: Degrees of Obesity. PD stages based on the E. P Classification 2018.
